# Congenital Esophageal Muscularis Propria Defect Found during Endoscopic Submucosal Dissection for Early Esophageal Cancer: A Case Report and Management

**DOI:** 10.1002/deo2.70274

**Published:** 2026-01-04

**Authors:** Ying Liu, Yuhan Ren, Fan Wang, Wei Cao, Yanqin Long

**Affiliations:** ^1^ Department of Gastroenterology Renmin Hospital Hubei University of Medicine Shiyan China; ^2^ Department of Gastroenterology The First Affiliated Hospital Zhejiang University School of Medicine Hangzhou China

**Keywords:** congenital, endoscopic submucosal dissection, esophageal, muscularis propria defects, perforation

## Abstract

Congenital esophageal muscularis propria defects are extremely rare, often complicating endoscopic submucosal dissection (ESD) for esophageal neoplasms. We report a 60‐year‐old man with early esophageal squamous cell carcinoma who underwent ESD. Intraoperatively, a congenital muscularis propria defect was incidentally identified as a translucent, respiration‐synchronized submucosal membrane, which exposed mediastinal structures after perforation. The defect was successfully managed via endoscopic endoclip‐nylon loop purse‐string closure. The patient recovered uneventfully, with pathologically confirmed negative margins and no diverticulum or stenosis at 3‐month follow‐up endoscopy. This case highlights that timely endoscopic recognition and management of such defects during ESD can avoid surgical intervention and ensure favorable outcomes, providing practical insights for endoscopists.

## 1 Introduction

Congenital esophageal muscularis propria defects are extremely rare, with only sporadic cases reported in Japan, most incidentally detected during endoscopic submucosal dissection (ESD) for superficial early esophageal neoplasms [1, 2, 3, 4, 5, 6, 7]. ESD carries elevated iatrogenic perforation risk in such cases, several previous cases required surgical intervention due to endoscopic management challenges. We report a successfully endoscopically managed case, highlighting key endoscopic features and the feasibility of conservative endoscopic treatment to guide practicing endoscopists.

## 2 Case Report

**FIGURE 1:**
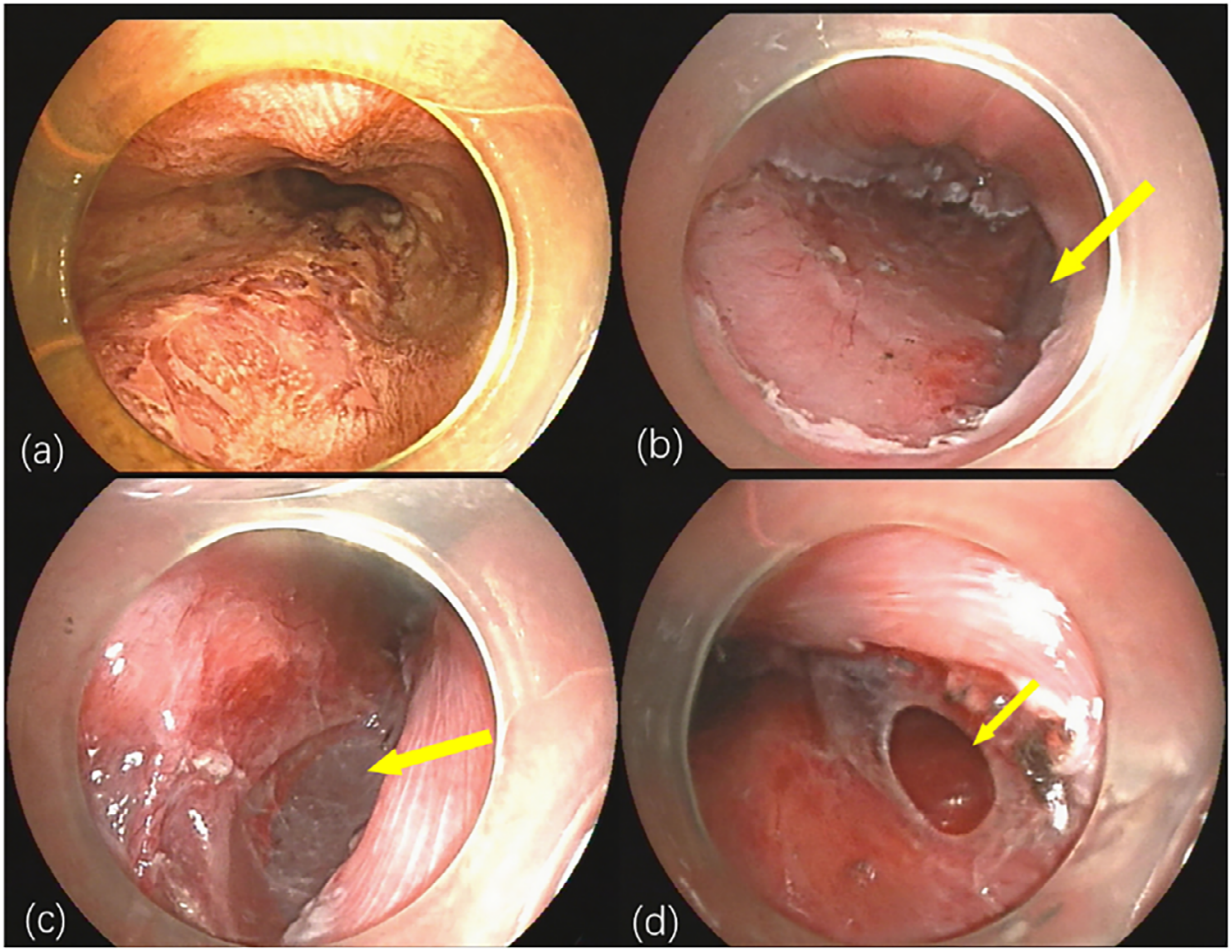
(a) Endoscopic view showing Lugol's iodine staining of the mucosal lesion in the mid‐thoracic esophagus (∼30 cm from incisors). The unstained area indicates suspicious mucosa. (b) Endoscopic view of the post‐endoscopic submucosal dissection (post‐ESD) wound bed following complete en bloc resection. Note the exposed submucosal layer and the muscularis propria defect (arrow). (c) A translucent membranous area in the submucosa was observed, pulsating synchronously with respiration (arrow). (d) Endoscopic view showing perforation of the translucent membrane (arrow) with complete absence of the muscularis propria layer. Mediastinal structures are visible through the defect.

**FIGURE 2:**
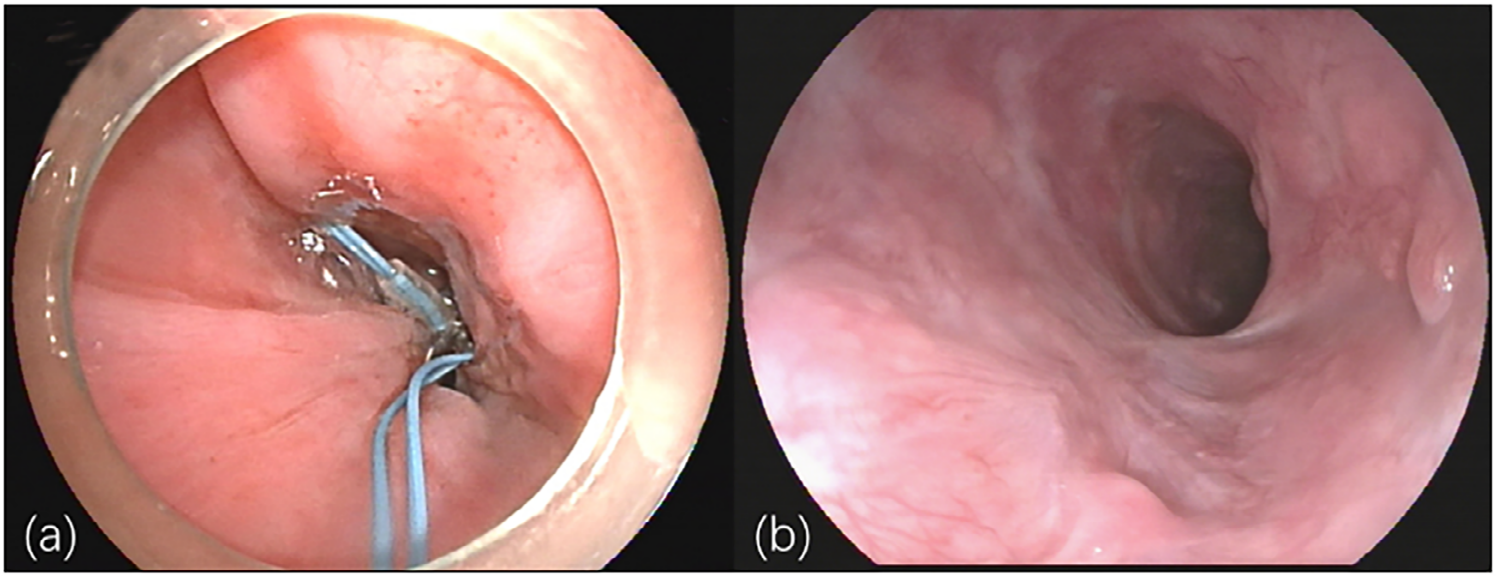
(a) Endoscopic closure of the perforation site: placement of endoclips at the defect margins, and complete sealing achieved by nylon loop purse‐string suture technique. (b) Three‐month postoperative endoscopic follow‐up showing scar formation at the healing site, with no evidence of esophageal diverticulum or stenosis.

A 60‐year‐old male farmer presented with dysphagia for over one month and had no history of previous illness, trauma, or surgery. Endoscopy revealed a 2‐cm diameter flat mucosal lesion (slight roughness and erythema) on the right side of the mid‐thoracic esophagus (∼30 cm from incisors), biopsy confirmed high‐grade intraepithelial neoplasia. Preoperative evaluations, including routine blood tests and chest computed tomography (CT), were unremarkable, with no esophageal diverticulum identified. Under general anesthesia, ESD was performed using a single‐channel endoscope (GIF‐Q260J; Olympus, Tokyo, Japan), a Dual knife (KD650; Olympus, Tokyo, Japan), and an electrosurgical generator (VIO 300D; ERBE Elektromedizin GmbH, Tübingen, Germany), with carbon dioxide (CO_2_) insufflation throughout the procedure. After Lugol's iodine staining (Figure 1a), marking, submucosal injection, and circumferential incision, the lesion (2.3 × 1.5 cm) was en bloc resected uneventfully. After specimen extraction, inspection of the wound surface revealed a 1‐cm diameter translucent submucosal membrane pulsating synchronously with respiration (Figure 1b,c and Video S1). The wound surface was neat, with no visible stumps of muscular layer injury. During inspection, the membrane was accidentally ruptured. Upon perforation of it (Figure 1d), an absence of the muscularis propria was noted, exposing mediastinal structures. A thoracic surgeon was consulted urgently, considering stable vital signs, endoscopic closure was attempted but failed with endoclips (Sureclip; Micro‐Tech Endoscopy, Nanjing) alone due to lesion size and high tension. Finally, a purse‐string suture was carried out with the same single‐channel endoscope. The nylon loop was secured around the wound with endoclips at 0.5 cm intervals, starting from the anal end. Clips were placed one by one on the right and left side, eight endoclips in total encircled the entire wound, tightening the loop achieved complete closure (Figure 2a). A jejunal feeding tube was subsequently placed.

Post‐ESD CT showed significant mediastinal and subcutaneous emphysema. The patient received cefoperazone sulbactam (3 g q12h, 10 days) and parenteral nutrition. Emphysema improved by postoperative day 3. Transient fever (peak 38.5°C), mild chest pain, and leukocytosis (white blood cell count elevated to 20,500/µL), resolved by postoperative day 8, with subsequent nasojejunal tube nutrition. Esophagography with Gastrografin (diatrizoate meglumine) on postoperative day 14 showed no contrast extravasation, allowing oral feeding and the nasojejunal tube removal. The patient was discharged on postoperative day 20 after full conservative recovery. Final pathology confirmed well‐differentiated superficial early esophageal squamous cell carcinoma (infiltration limited to the lamina propria) with negative margins, and muscularis propria was not visualized in the specimen. Three‐month follow‐up endoscopy showed scar formation without diverticulum or stenosis (Figure 2b).

## 3 Discussion

Congenital esophageal muscularis propria defects are exceedingly rare, with few cases identified during ESD [1, 2, 3, 4, 5, 6, 7]. Several compromise complete resections or require surgical intervention [3, 4]. A 2013–2019 retrospective study of esophageal ESD across 10 Japanese centers reported a 0.29% (5/1708) incidence of segmental muscularis absence [7]. Different from previous reports, this defect was initially overlooked due to uneventful ESD progress. After complete lesion resection and specimen retrieval, a translucent, respiration‐synchronized membrane lacking a muscular layer was noted, inadvertently perforated due to limited clinical experience. Prompt endoscopic purse‐string suturing ensured successful closure; the patient recovered fully with conservative treatment and no stricture. Notably, all previously documented cases were from Japan, with perforation typically arising during lesion dissection, managed via immediate suture followed by traction‐assisted or snare resection. To our knowledge, this is the first reported case in China and outside Japan. Enhanced awareness may facilitate more global diagnoses, and preoperative or intra‐ESD recognition could help reduce similar perforation risks.

In this case, general anesthesia ensured hemodynamic stability and procedural safety throughout ESD. Studies indicate that compared with conscious sedation, general anesthesia reduces esophageal ESD‐related complications (e.g., arrhythmia, aspiration hypoxemia, bleeding) and perforation risk by suppressing patient movement and stabilizing respiration, yielding superior clinical outcomes [8]. It is the standard approach in most Chinese endoscopic centers, except those with contraindications. Intraoperative CO_2_ insufflation also facilitated rapid recovery by alleviating mediastinal emphysema. CO_2_ is rapidly absorbed via the gastrointestinal tract and excreted through expiration, and multiple studies have confirmed its safety and efficacy as an alternative to air insufflation [9].

ESD‐related iatrogenic perforations most commonly result from electrical damage due to improper endoknife use on the muscularis propria, typically presenting as small muscular defects. Recent studies have reported large muscular defects during esophageal ESD without iatrogenic muscular injury—segmental absence of intestinal musculature in adults [1, 2, 3, 4, 5, 6, 7], characterized by well‐defined circular perforation, complete muscularis propria deficiency, and no periforational hemorrhage or necrosis [10]. In our case, no muscular layer electrical injury occurred, and dissection proceeded smoothly. The focal muscularis propria defect was identified only after en bloc lesion resection; the wound was clean without muscular stumps, and no muscle tissue was found in the resected specimen. Consistent with previous reports [1, 2, 3, 4, 5, 6, 7], this defect was confirmed as pre‐existing segmental esophageal muscularis absence, rather than an ESD‐induced injury.

Esophageal muscularis propria defects are extremely rare, with only a few case reports, and their exact pathogenesis remains unclear. Segmental muscularis absence has two recognized etiological subtypes: congenital and acquired [7]. Congenital cases are attributed to incomplete segmental muscular layer development, while acquired cases are speculated to be associated with ischemia‐induced fibrosis and chronic inflammation, muscle fiber degeneration (e.g., autoimmune connective tissue disease [ACTD]), and Ehlers‐Danlos syndrome comorbidity, though none of these have been confirmed [10]. In this case, no significant submucosal fibrosis was observed intraoperatively. The patient had no history of surgery, trauma, or periesophageal inflammatory conditions (e.g., tuberculosis, histoplasmosis, malignancy, and ACTD), and CT findings were unremarkable. These features support a presumptive diagnosis of congenital esophageal muscularis propria defect, consistent with previous reports [7]. Another condition requiring differential diagnosis is pseudodiverticulum; pulsion diverticula (e.g., Zenker's diverticulum and supradiaphragmatic diverticulum) may mimic muscular defects via mucosal and submucosal protrusion through the muscular layer. Such lesions are typically identifiable as mucosal depressions on endoscopy or CT. However, our patient had no mucosal depression, nor periesophageal inflammation, fibrosis, or adhesions suggestive of a traction diverticulum. Nevertheless, long‐term elevated esophageal pressure may potentially lead to pulsion diverticulum formation at the muscular defect site, a possibility that cannot be excluded.

Segmental muscularis propria absence is difficult to predict preoperatively [1, 2, 3, 4, 5, 6, 7]. Theoretically, endoscopy ultrasound (EUS) and CT might indicate such defects, but EUS is not routinely used for preoperative assessment of superficial esophageal lesions. Although preoperative EUS could be considered to facilitate postoperative analysis of such special conditions, congenital esophageal muscularis propria defects are extremely rare; thus, considering cost‐effectiveness and risk‐benefit balance, performing EUS solely to detect this defect is not recommended. Preoperative CT is standard for esophageal ESD patients at our center. In this case, postoperative joint re‐evaluation of CT images with radiologists showed no obvious abnormalities, such as esophageal thinning or localized rigidity. We speculate this may be due to the small defect size, absence of surface depression, longitudinal non‐circumferential distribution, and esophageal contractile state. Further investigation requires more clinical case accumulation.

Immediate closure is crucial once perforation develops at a muscular defect during esophageal ESD. The closure method is similar to that for conventional iatrogenic perforations, but the key difference is that closure should cover the entire muscular defect (not just the perforation site) to prevent recurrence. Endoclips can be used for closure [1, 2]; when clipping is challenging, purse‐string suturing, as in this case, is an alternative. Other effective methods reported include polyglycolic acid sheets with fibrin glue, over‐the‐scope clips, and stents, but these require specialized devices or reagents. We thus opted for the endoclip‐nylon loop approach using standard clinical tools. In addition to prompt closure, intensive postoperative monitoring, antibiotics, and nutritional support were critical for successful conservative treatment.

In conclusion, a congenital esophageal muscularis propria defect is an extremely rare condition, typically diagnosed incidentally during esophageal ESD. Endoscopists performing this procedure should remain vigilant to this possibility. Early detection of the defect enabled us to perform shallow submucosal dissection, which likely avoided esophageal perforation. Once perforation occurs, immediate suturing using endoclips or combined with a nylon loop contributes to the success of conservative treatment.

## Author Contributions

**Ying Liu**: endoscopy performance; drafting the manuscript. **Yuhan Ren**: inpatient management; ward care supervision. **Fan Wang**: endoscopic assistance; technical support during endoscopy. **Wei Cao**: postoperative patient follow‐up. **Yanqin Long**: Study conception and design; drafting the manuscript; critical revision; final approval of the manuscript.

## Conflicts of Interest

The authors declare no conflicts of interest.

## Funding

No specific funding was received for the study.

## Ethics Statement

Ethical approval was not required for this case report, given its nature as a medical activity.

## Supporting information




**VIDEO S1**: Congenital esophageal muscularis propria defect found during endoscopic submucosal dissection for early esophageal cancer.
